# *orf137* triggers cytoplasmic male sterility in tomato

**DOI:** 10.1093/plphys/kiac082

**Published:** 2022-02-25

**Authors:** Kosuke Kuwabara, Shin-ichi Arimura, Kenta Shirasawa, Tohru Ariizumi

**Affiliations:** 1 Graduate School of Life and Environmental Sciences, University of Tsukuba, Tsukuba, Ibaraki 305-8577, Japan; 2 Japan Society for the Promotion of Science (JSPS), Kojimachi, Tokyo 102-0083, Japan; 3 Graduate School of Agricultural and Life Sciences, University of Tokyo, Bunkyo, Tokyo 113-8654, Japan; 4 Kazusa DNA Research Institute, Kisarazu, Chiba 292-0818, Japan; 5 Tsukuba Plant Innovation Research Center, Tsukuba, Ibaraki 305-8577, Japan

## Abstract

mitoTALEN, a mitochondrial genome editing technology, demonstrated that the mitochondrial gene *orf137* is responsible for inducing cytoplasmic male sterility in tomato.

Dear Editor,

Cytoplasmic male sterility (CMS) refers to the inability of a plant to produce fertile pollen due to nuclear and mitochondrial genomic incompatibility. The mechanism that triggers CMS has been explored in several cereal crops and occurs in the presence of CMS-associated genes encoded by the mitochondrial genome; however, in tomato (*Solanum lycopersicum*), this mechanism remains unclear (Chen and Liu, 2014). To date, CMS-associated genes have been identified in various crops such as rice (*Oryza sativa*), wheat (*Triticum aestivum*), sugar beet (*Beta vulgaris*), carrot (*Daucus carota*), radish (*Raphanus sativus*), rapeseed (*Brassica napus*), and maize (*Zea mays*) (Chen and Liu, 2014), but not in tomato. Recently, mitochondrial transcription activator-like effector nucleases (mitoTALENs) have emerged as a revolutionary genome editing tool enabling the targeted disruption of genes in the mitochondrial genome. mitoTALENs have been used to disrupt the mitochondrial CMS-associated genes *open reading frame 79* (*orf79*) and *orf352* in rice and *orf125* in rapeseed; furthermore, the pollen fertility of the resulting edited plants was restored, thus revealing the role of CMS-associated genes in male sterility (Kazama et al., 2019; Omukai et al., 2021). However, CMS-associated genes have not been identified in tomato.

In this study, CMS tomato, previously generated by asymmetric cell fusion between the potato wild relative *Solanum acaule* as the cytoplasmic donor and *S. lycopersicum* as the nuclear donor, was used (Melchers et al., 1992). Previously, we assembled mitochondrial genomes of CMS and fertile tomato cultivars using PacBio long-read sequencing and annotated their ORFs (Kuwabara et al., 2021). By comparing their ORFs, a candidate CMS-associated gene, *orf137*, specifically present in the CMS tomato mitochondrial genome, was identified. The *orf137* gene encodes 137 amino acids that are expressed in tomato anthers and pollen (Kuwabara et al., 2021) and demonstrates mild sequence similarity to the candidate CMS-associated gene (*orf507*) of CMS pepper (*Capsicum annuum*) (Gulyas et al., 2010); however, the association between *orf137* and CMS remains to be elucidated. To address this, a mitoTALEN vector (*mTAL137*) was constructed (Supplemental Text), in combination with TALEN editing, with mitochondrial localization signals that targeted the *orf137*-coding sequence for disruption to investigate whether *orf137* is responsible for the CMS trait in tomato (Figure 1A). Thereafter, the *mTAL137* transformation vector was introduced into the CMS tomato, previously named Dwarf “CMS[P]”, whose genetic background is the dwarf cultivar “Micro-Tom” (Kuwabara et al., 2021), via the *Agrobacterium tumefaciens* transformation method. Thus, we obtained three independent transgenic lines: *mTAL137 #1*, *#2*, and *#3*.

**Figure 1:**
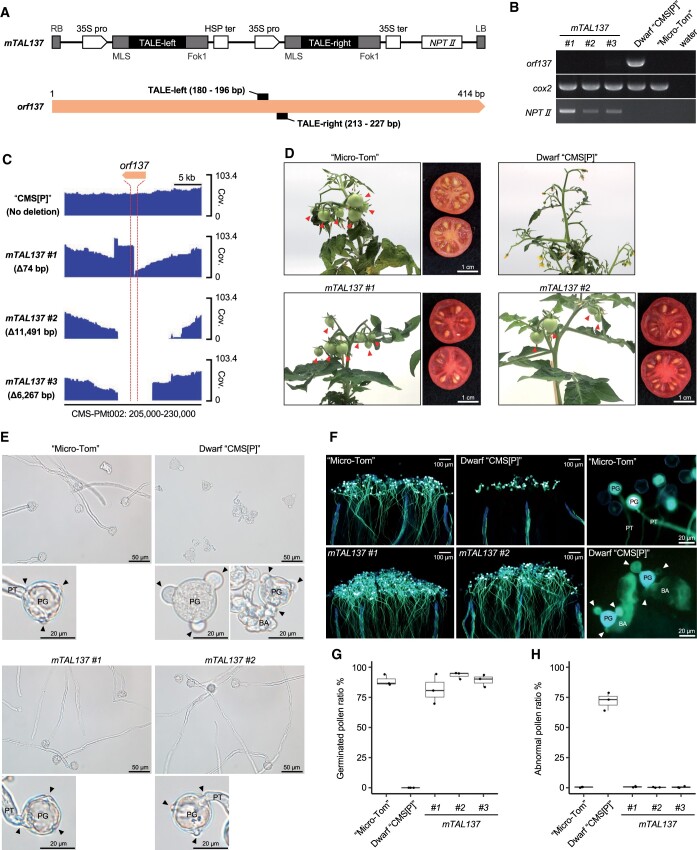
*orf137* triggers cytoplasmic male sterility in tomato. A, *mTAL137* construct and binding sites of each TALEN in the *orf137* sequence. LB, left border; RB, right border; 35S pro, cauliflower mosaic virus (CaMV) 35S promoter; 35S ter, CaMV terminator; HSP ter, Arabidopsis heat shock protein terminator; MLS, mitochondrial localization signal. B, PCR analysis of *orf137* in *mTAL137* T_0_ lines. “Micro-Tom”, which does not have the *orf137* gene, and water served as negative controls; *cytochrome oxidase subunit 2* (*cox2*) served as the mitochondrial genome control; and *neomycin phosphotransferase II* (*NPTII*) served as the marker gene for the *mTAL137* vector. C, Long-read sequencing coverage of *orf137* and its vicinity (CMS-PMt002: 205,000–230,000 bp; accession number LC613119 in the DDBJ database) in *mTAL137* T_0_ lines. The location of *orf137* is indicated by the red dashed lines. The read coverage of “CMS[P]”, in which the *mTAL137* construct was not introduced, was obtained from our previous report (Kuwabara et al., 2021). Cov., read coverage. D, Fruit and seed formation in *mTAL137* T_0_ lines. Red arrows indicate the fruits on the plants. E, Phenotypes of pollen after 4 h of incubation in germination media for “Micro-Tom”, Dwarf “CMS[P]”, and *mTAL137* T_1_ lines. Black arrows indicate the positions of apertures. PG, pollen grain; PT, pollen tube; BA, burst aperture. F, Phenotypes of pollen stained with aniline blue 24 h after pollination on stigma for “Micro-Tom”, Dwarf “CMS[P]”, and *mTAL137* T_1_ lines. White arrows indicate the positions of apertures. PG, pollen grain; PT, pollen tube; BA, burst aperture. G, Germinated pollen ratio 4 h after incubation in germination media for “Micro-Tom”, Dwarf “CMS[P]”, and *mTAL137* T_1_ lines. The boxplots represent interquartile ranges, the black center lines indicate the medians, and the whiskers represent 1.5 × interquartile ranges. Each point indicates the value for each experiment. Values were calculated from three independent experiments (*n* = 3). H, Abnormal pollen ratio 4 h after incubation in germination media for “Micro-Tom”, Dwarf “CMS[P]”, and *mTAL137* T_1_ lines. The boxplots represent interquartile ranges, the black center lines indicate the medians, and the whiskers represent 1.5 × interquartile ranges. Each point indicates the value for each experiment. Values were calculated from three independent experiments (*n* = 3).

First, genomic PCR analysis was performed to assess the genomic region around the target site using primers flanking the coding region of *orf137.* However, the polymerase chain reaction (PCR) fragment including *orf137*, which was amplified in Dwarf “CMS[P]”, was not amplified in any of the *mTAL137* T_0_ lines (Figure 1B), indicating that the genomic regions around *orf137* were modified by mitoTALEN. To examine the precise genome structure around *orf137*, long-read sequences were obtained from the transgenic plants using the PacBio system and mapped on the previously constructed mitochondrial reference genome of “CMS[P]” (CMS-PMt002, Kuwabara et al., 2021), after which the status of read coverage was visualized using the Integrative Genomics Viewer (Figure 1C). Unlike the “CMS[P]” control, we observed deletions of 74 bp, 11,491 bp, and 6,267 bp around the target site in the *mTAL137 #1*, *#2*, and *#3* T_0_ lines, respectively (Figure 1C). This indicated that mitoTALEN induced targeted mutagenesis, causing double-stranded breaks (DSBs) around *orf137*; only *orf137* was present within the deleted regions of the *mTAL137* T_0_ lines (Supplemental Figure S1).

Next, we explored the repair mechanism of DSBs created by mitoTALEN. In a previous study reporting the disruption of CMS-associated genes in rice and rapeseed by mitoTALEN, the DSBs were followed by homologous recombination events (Kazama et al., 2019; Omukai et al., 2021). We extracted long sequence reads covering the deleted genomic regions and used them to assess the sequence of the boundary genomic region after DSBs. All collected long reads from the three *mTAL137* T_0_ lines included recombination sequences joined between the free ends and other homologous mitochondrial genome regions. For example, 746 bp of the left-side free ends in both *mTAL137 #2* and *#3* T_0_ lines were at positions 214,004–214,749 of the reference genome CMS-PMt002, which were recombined with a 726-bp genomic region at positions 41,249–41,974 of the reference genome. Moreover, 8 bp of the right-side free ends at positions 226,241–226,248 in *mTAL137 #2* and 51 bp of that at 221,017–221,067 in *mTAL137 #3* were recombined with 8 bp at positions 226,439–226,432 and 52 bp at 155,367–155,316 of the reference, respectively. Similar homologous recombination events were observed for *mTAL137 #1* (Supplemental Figure S2)*.* These recombination sequences of *mTAL137* T_0_ lines were confirmed by Sanger sequencing of PCR products using (Supplemental Table S1) primers that specifically amplified the recombination sequences; the results were consistent with the analysis using long sequence reads, as mentioned earlier (Supplemental Figure S3). These results indicate that DSBs of *orf137*-deleted CMS tomatoes are repaired through homologous recombination using homologous sequences as templates.

The growth of the transgenic plants was similar to that of the wild-type “Micro-Tom”, and mature fruits full of seeds were produced (Figure 1D). To assess the pollen viability of the transgenic plants, we used T_1_ plants derived from the self-pollination of *mTAL137 #1*, *#2*, and *#3* T_0_ plants and confirmed that none of the *mTAL137* T_1_ plants contained *orf137* (Supplemental Figure S4). No difference was observed in the appearance of the “Micro-Tom” and Dwarf “CMS[P]” pollen before incubation in the germination media, and three germination apertures (i.e. germination pores) were observed in both (Supplemental Figure S5). However, the Dwarf “CMS[P]” pollen started showing an abnormal phenotype with expanded apertures, and some of the pollen burst after 4-h incubation in the germination media (Figure 1E). This expanded aperture phenotype was also observed in pollen on the stigma after 24-h pollination (Figure 1F). A high rate (∼72%) of abnormal pollen phenotypes was observed in Dwarf “CMS[P]”, from which no pollen germinated in the germination media or on the stigma (Figure 1, G and H). In contrast, pollen phenotypes of the three *mTAL137* T_1_ lines resembled those of “Micro-Tom” (Figure 1, E and F), and the pollen of all four varieties was generally normal (<1% abnormal). Pollen of the *mTAL137 #1*, *#2*, and *#3* T_1_ plants germinated at rates of 82%, 93%, and 89%, respectively, which were comparable to the 89% germination rate of “Micro-Tom” (Figure 1, E–G) and indicated that the viability of the pollen was fully restored in the transgenic lines.

In summary, this study shows that mitoTALENs function in tomato, and the repair mechanism following DSBs is mediated by homologous recombination, similar to that of rice, *Brassica*, and Arabidopsis (*Arabidopsis thaliana*) (Kazama et al., 2019; Arimura et al., 2020; Omukai et al., 2021). This study also showed that mitochondrial *orf137* is the CMS-associated gene that is completely responsible for the male sterile phenotype of CMS tomato. Our results provide a foundation to develop an efficient F_1_ hybrid breeding system using CMS tomato carrying *orf137*.

## Supplemental data

The following materials are available in the online version of this article.

**Supplemental Table S1.** Oligonucleotide sequences of PCR primers.

**Supplemental Figure S1.** Mitochondrial gene locations around the region of *orf137*.

**Supplemental Figure S2.** Homologous recombination to repair DSBs generated by mitoTALEN.

**Supplemental Figure S3.** Verification of recombination sequences in *mTAL137* T_0_ lines.

**Supplemental Figure S4.** PCR analysis of *mTAL137* T_1_ plants.

**Supplemental Figure S5.** Pollen phenotype before incubation in germination media.

**Supplemental text.** Materials and Methods in this study.

## Supplementary Material

kiac082_Supplementary_Data

## Notes

T.A. designed the project, S.A. created the transformation vector; K.K. generated and characterized the transgenic plants; K.S. performed the genome analysis; T.A., K.K., K.S., and S.A. wrote the manuscript.

The author responsible for distribution of materials integral to the findings presented in this article in accordance with the policy described in the Instructions for Authors (https://academic.oup.com/plphys/pages/general-instructions) is: Tohru Ariizumi (ariizumi.toru.ge@u.tsukuba.ac.jp).
