# Supplementary material for: orf137 triggers cytoplasmic male sterility in tomato
Source: Plant Physiol. 2022 Feb 25;189(2):465–8. doi: 10.1093/plphys/kiac082 (PMC9157052; doi:10.1093/plphys/kiac082)
Supplement: kiac082_Supplementary_Data [file kiac082_supplementary_data.zip › 20220301_Kuwabara_Supplementary_Table_Figures.pdf]

Supplementary Table S1. Oligonucleotide sequences of PCR primers.

Primer name	Sequence (5'-3')	Purpose
*orf137*_F	CGATTGAGAAAGCGGCAGGC	Amplification of *orf137*
*orf137*_R	GTTATTTTCGCTGCAACGGCG	
*cox2*_F	CCCGCAAAGGATTGTTCATGG	Amplification of *cox2*
*cox2*_R	CGTATAGGGCTCTTTGCTGGTAG	
*NPTII*_F	ATGATTGAACAAGATGGATTGCAC	Amplification of *NPT II*
*NPTII*_R	TCAGAAGAACTCGTCAAGAAGGCG	
p1_Left_F	ACAGGAGAAGGCCGCAATAC	Amplification of recombination sequence that were joined between
p1_Left_R	GTTATTTTCGCTGCAACGGCG	the left-side free ends and new connected region in *mTAL137 #1* line
p1_Right_F	CTCGACCTTAACTCGATCGCC	Amplification of recombination sequence that were joined between
p1_Right_R	CGACTATGGTACATCGCTTTCTC	the right-side free ends and new connected region in *mTAL137 #1* line
p2,3_Left_F	TGAATAGGCATGTGGGAACAGC	Amplification of recombination sequence that were joined between
p2,3_Left_R	GCTTTCTTGCTCCTCTCCTTAGA	the left-side free ends and new connected region in *mTAL137 #2* and *#3* lines
p2_Right_F	CGCAAAAGGCTATTGAAGGGGC	Amplification of recombination sequence that were joined between
p2_Right_R	CTGCAATAGCCCGACGAC	the right-side free ends and new connected region in *mTAL137 #2* lines
p3_Right_F	GTCAATATTGGCGCCCTTACCA	Amplification of recombination sequence that were joined between
p3_Right_R	CAAGAATACTCATCCGAGCCGCA	the right-side free ends and new connected region in *mTAL137 #3* line

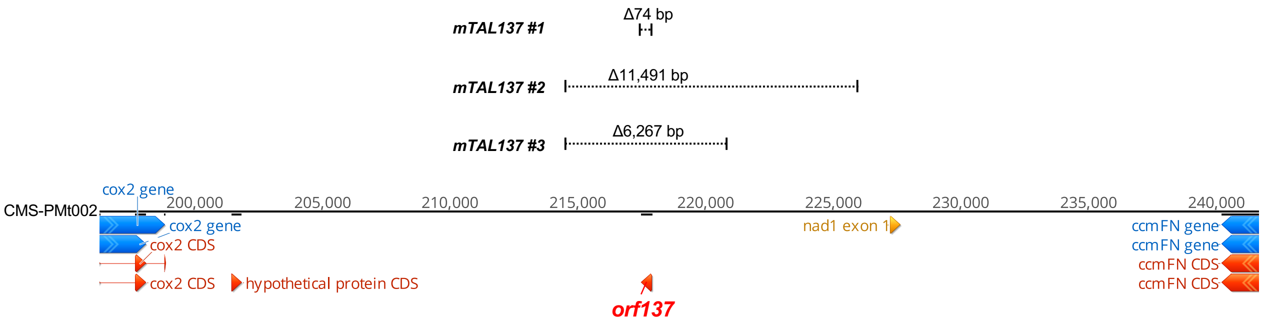

Supplementary Figure S1. Mitochondrial gene locations around the region of *orf137*.

Mitochondrial genome of CMS tomato (CMS-PMt002; accession number LC613119 in DDBJ database) is annotated by a software Geneious (Biomatters) using reference mitochondrial genomes (GenBank accession number MF034192, MF034193, NC_035964, MF989953-MF989957, MN104801-MN104803 and MN114537-MN114539). Black dashed lines indicate the locations of deleted regions in *mTAL137* T_0 lines. Colored triangles and pentagons indicate the positions of mitochondrial genes. *cox2*, *cytochrome oxidase subunit 2*; *nad1*, *NADH dehydrogenase subunit 1*; *ccmFN*, *cytochrome c maturation subunit FN*; CDS, coding sequence.

mTAL137 #1

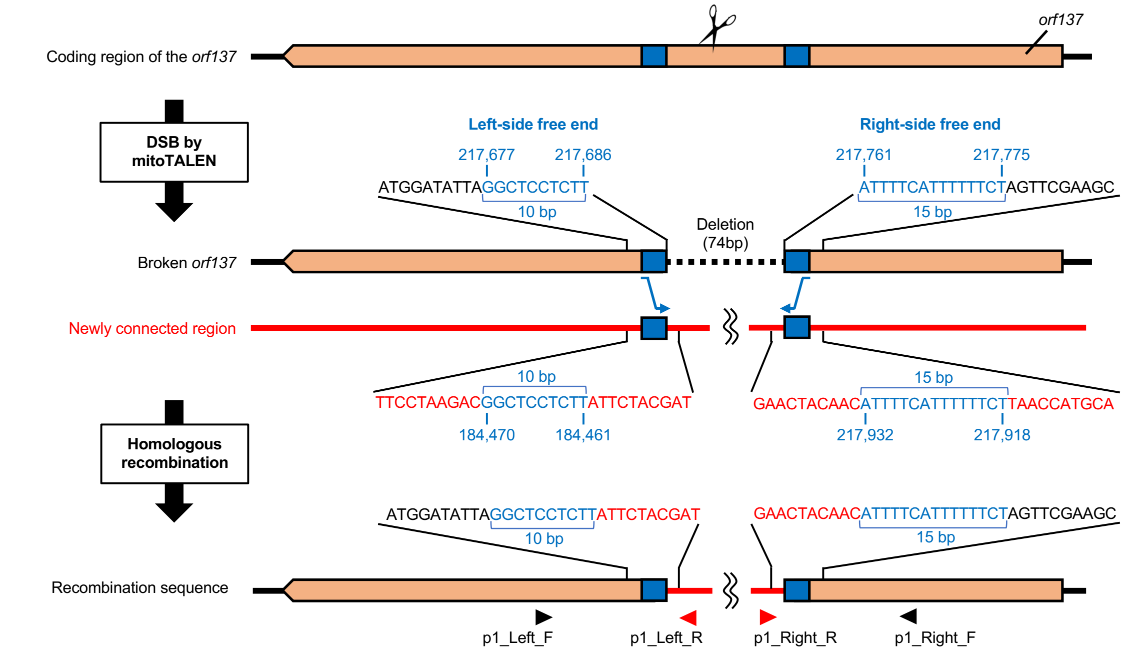

mTAL137 #2

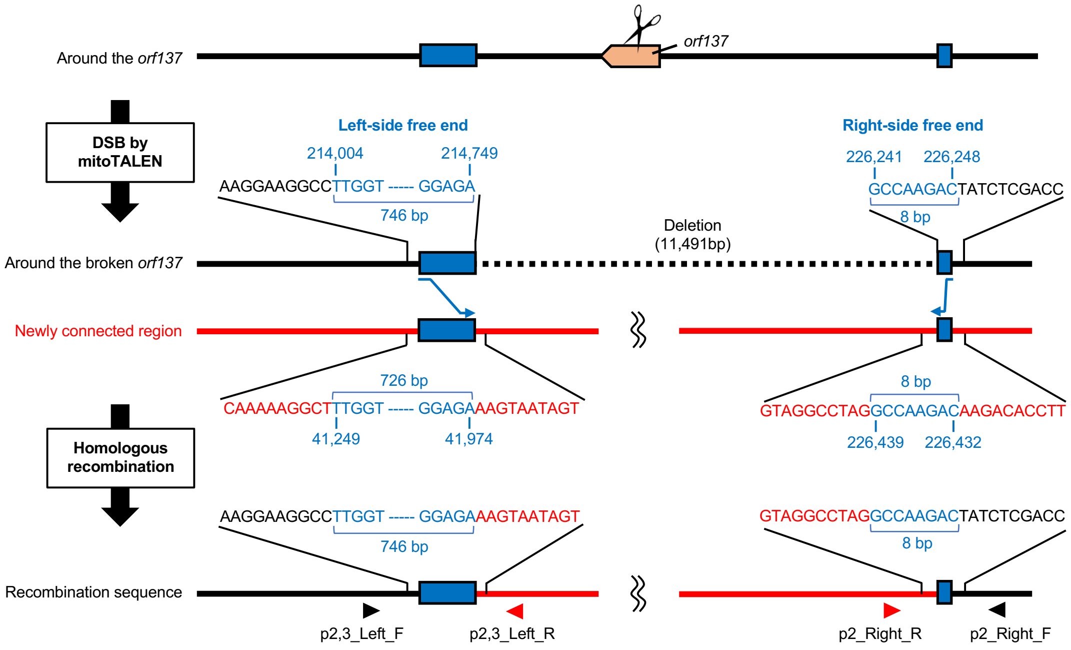

***mTAL137* #3**

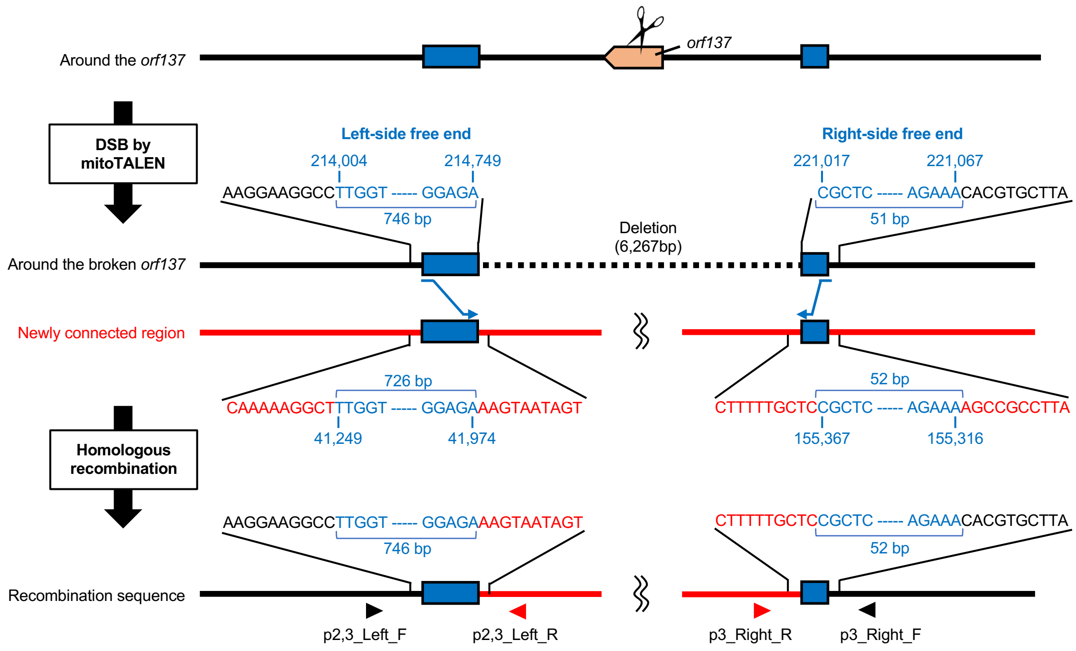

Supplementary Figure S2. Homologous recombination to repair DSBs generated by mitoTALEN.

Schematic drawings of predicted homologous recombination events in *mTAL137* T_0 plants. Scissors indicate the positions for target of mitoTALEN. Horizontal black lines indicate the coding region or around *orf137*, and horizontal red lines indicate the regions to which left or right-side free ends newly connected. Dashed lines indicate the deleted regions. Blue boxes indicate the homologous sequences which exist around deleted regions and newly connected regions. Black, red and blue letters on the DNA sequences correspond to horizontal black and red lines or blue boxes, respectively. Blue numbers indicate the end points of homologous sequences on the reference genome CMS-PMt002 (DDBJ accession number LC613119). Blue arrows indicate the process of homologous recombination using homologous sequences as templates. Black or red arrowheads indicate the positions of primers just before deleted regions or on newly connected regions, respectively, which used for amplification of recombination sequences in *mTAL137* T_0 lines. DSB, double-stranded break.

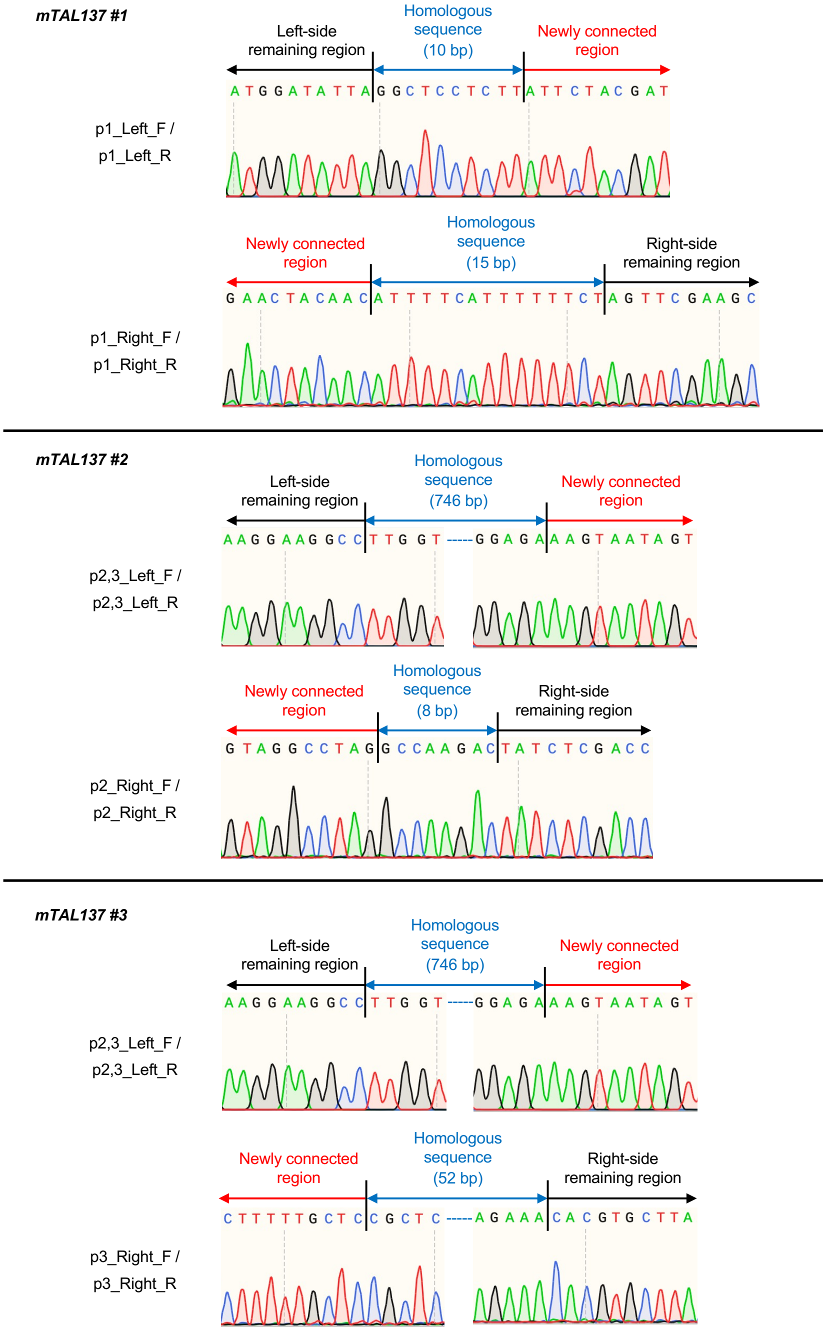

Supplementary Figure S3. Verification of recombination sequences in *mTAL137* T_0 lines.

DNA sequencing profiles of the recombination sequences in *mTAL137* T_0 lines. The positions of primer used for PCR analysis and sanger-sequencing are shown in the schematic drawings of Supplementary Figure S2.

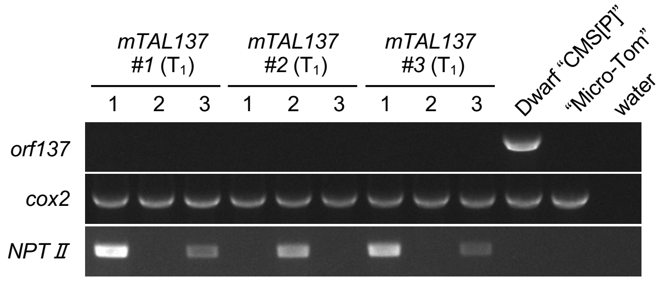

Supplementary Figure S4. PCR analysis of *mTAL137* T_1 plants.

PCR analysis of *orf137* in *mTAL137* T_1 lines. "Micro-Tom" which doesn't have *orf137* and water are negative control. *cox2* (*cytochrome oxidase subunit 2*) is a control of mitochondrial genome and *NPT II* (*neomycin phosphotransferase II*) is a marker gene of the *mTAL137* vector.

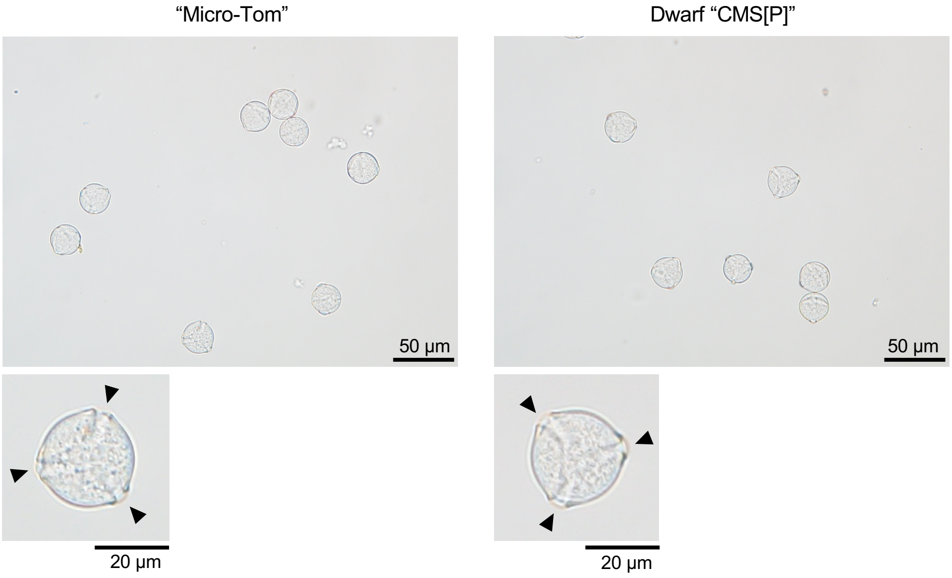

Supplementary Figure S5. Pollen phenotype before incubation in germination media.

Appearance of pollen before incubation in germination media for “Micro-Tom” and Dwarf “CMS[P]”. Black arrows indicate the positions of aperture.
