# Supplementary material for: orf137 triggers cytoplasmic male sterility in tomato
Source: Plant Physiol. 2022 Feb 25;189(2):465–8. doi: 10.1093/plphys/kiac082 (PMC9157052; doi:10.1093/plphys/kiac082)
Supplement: kiac082_Supplementary_Data [file kiac082_supplementary_data.zip › 20220301_Kuwabara_Supplementary_Table_Figures.pptx]

## Slide 1
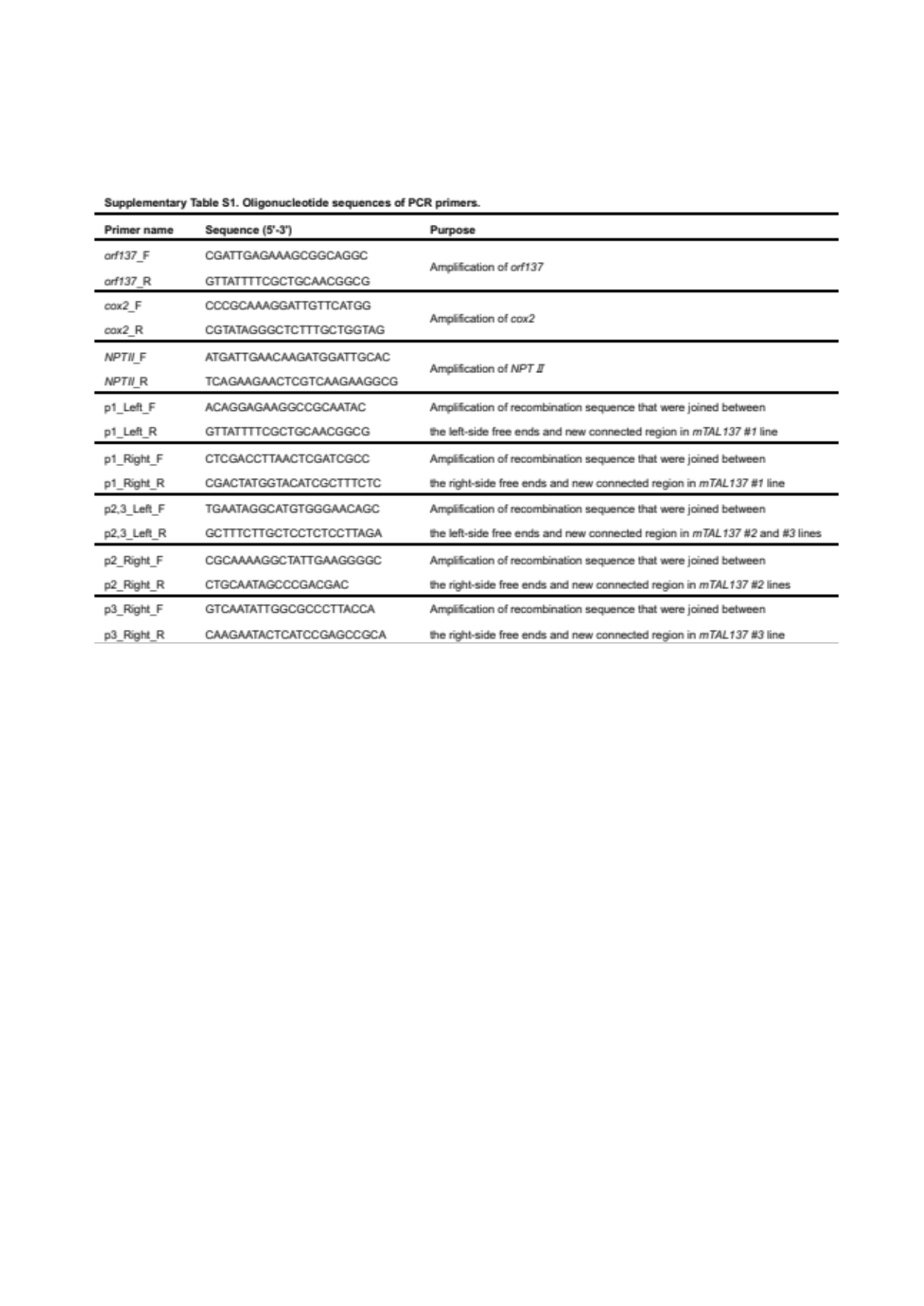

## Slide 2
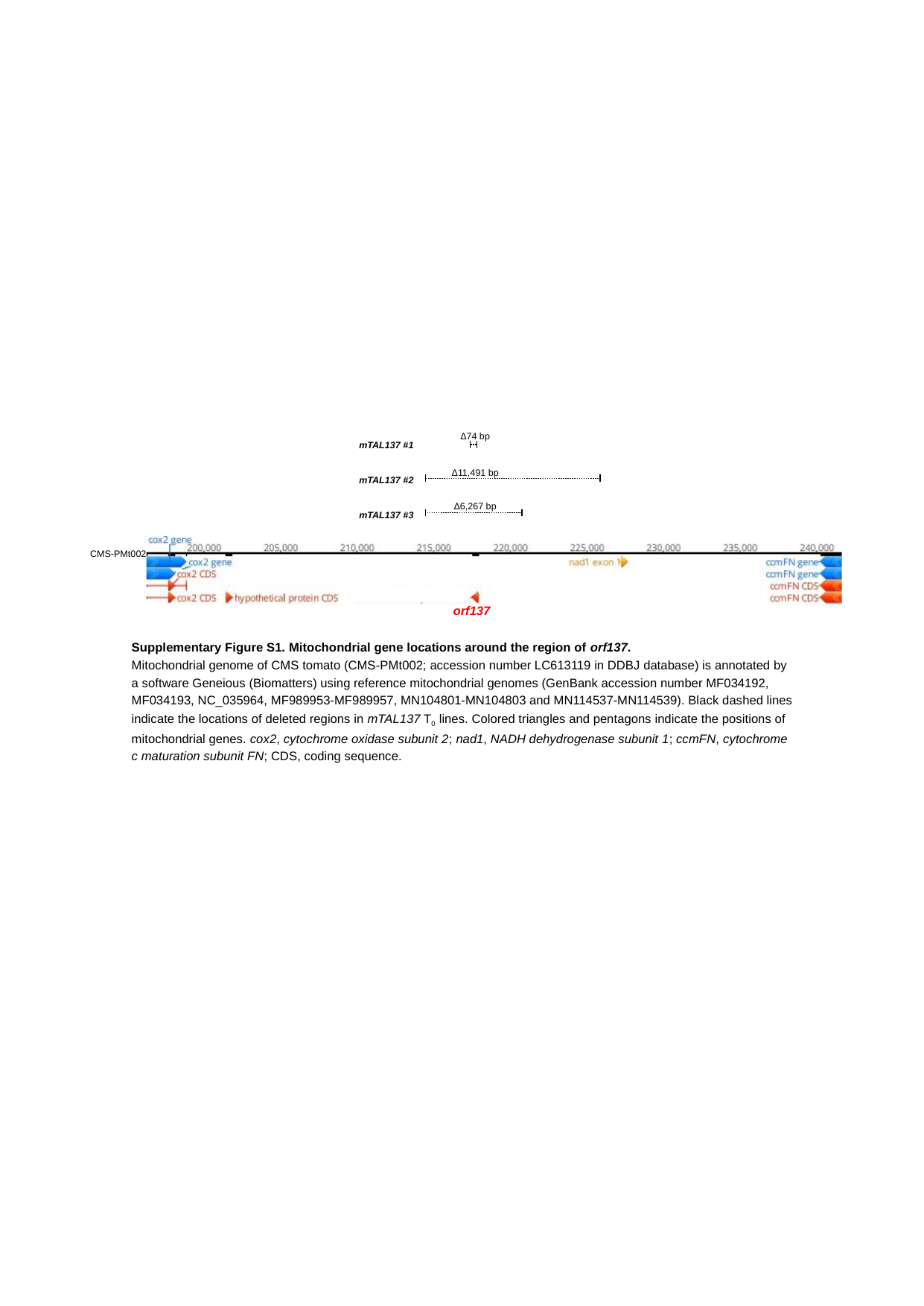

Δ74 bp
mTAL137 #1
Δ11,491 bp
mTAL137 #2
Δ6,267 bp
mTAL137 #3
CMS-PMt002
orf137
Supplementary Figure S1. Mitochondrial gene locations around the region of orf137.
Mitochondrial genome of CMS tomato (CMS-PMt002; accession number LC613119 in DDBJ database) is annotated by a software Geneious (Biomatters) using reference mitochondrial genomes (GenBank accession number MF034192, MF034193, NC_035964, MF989953-MF989957, MN104801-MN104803 and MN114537-MN114539). Black dashed lines indicate the locations of deleted regions in mTAL137 T0 lines. Colored triangles and pentagons indicate the positions of mitochondrial genes. cox2, cytochrome oxidase subunit 2; nad1, NADH dehydrogenase subunit 1; ccmFN, cytochrome c maturation subunit FN; CDS, coding sequence.

## Slide 3
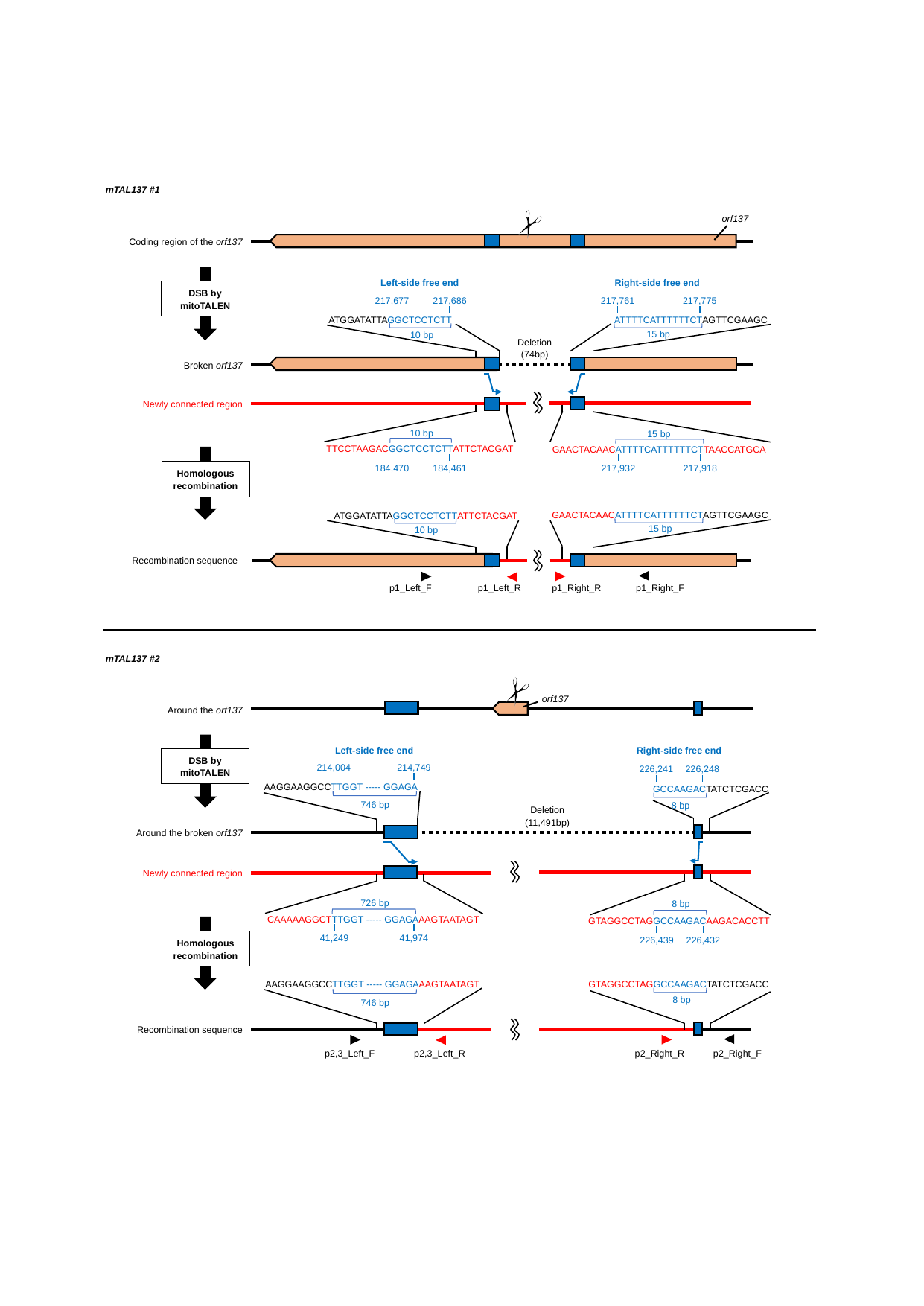

mTAL137 #1
orf137
Coding region of the orf137
Left-side free end
Right-side free end
DSB by
mitoTALEN
217,677
217,686
217,761
217,775
ATGGATATTAGGCTCCTCTT
ATTTTCATTTTTTCTAGTTCGAAGC
15 bp
10 bp
Deletion
(74bp)
Broken orf137
Newly connected region
10 bp
15 bp
TTCCTAAGACGGCTCCTCTTATTCTACGAT
GAACTACAACATTTTCATTTTTTCTTAACCATGCA
184,470
184,461
217,932
217,918
Homologous
recombination
GAACTACAACATTTTCATTTTTTCTAGTTCGAAGC
ATGGATATTAGGCTCCTCTTATTCTACGAT
15 bp
10 bp
Recombination sequence
p1_Left_F
p1_Left_R
p1_Right_F
p1_Right_R
mTAL137 #2
orf137
Around the orf137
Left-side free end
Right-side free end
DSB by
mitoTALEN
214,004
214,749
226,241
226,248
AAGGAAGGCCTTGGT ----- GGAGA
GCCAAGACTATCTCGACC
746 bp
8 bp
Deletion
(11,491bp)
Around the broken orf137
Newly connected region
726 bp
8 bp
CAAAAAGGCTTTGGT ----- GGAGAAAGTAATAGT
GTAGGCCTAGGCCAAGACAAGACACCTT
41,249
41,974
226,439
226,432
Homologous
recombination
GTAGGCCTAGGCCAAGACTATCTCGACC
AAGGAAGGCCTTGGT ----- GGAGAAAGTAATAGT
8 bp
746 bp
Recombination sequence
p2,3_Left_F
p2,3_Left_R
p2_Right_R
p2_Right_F

## Slide 4
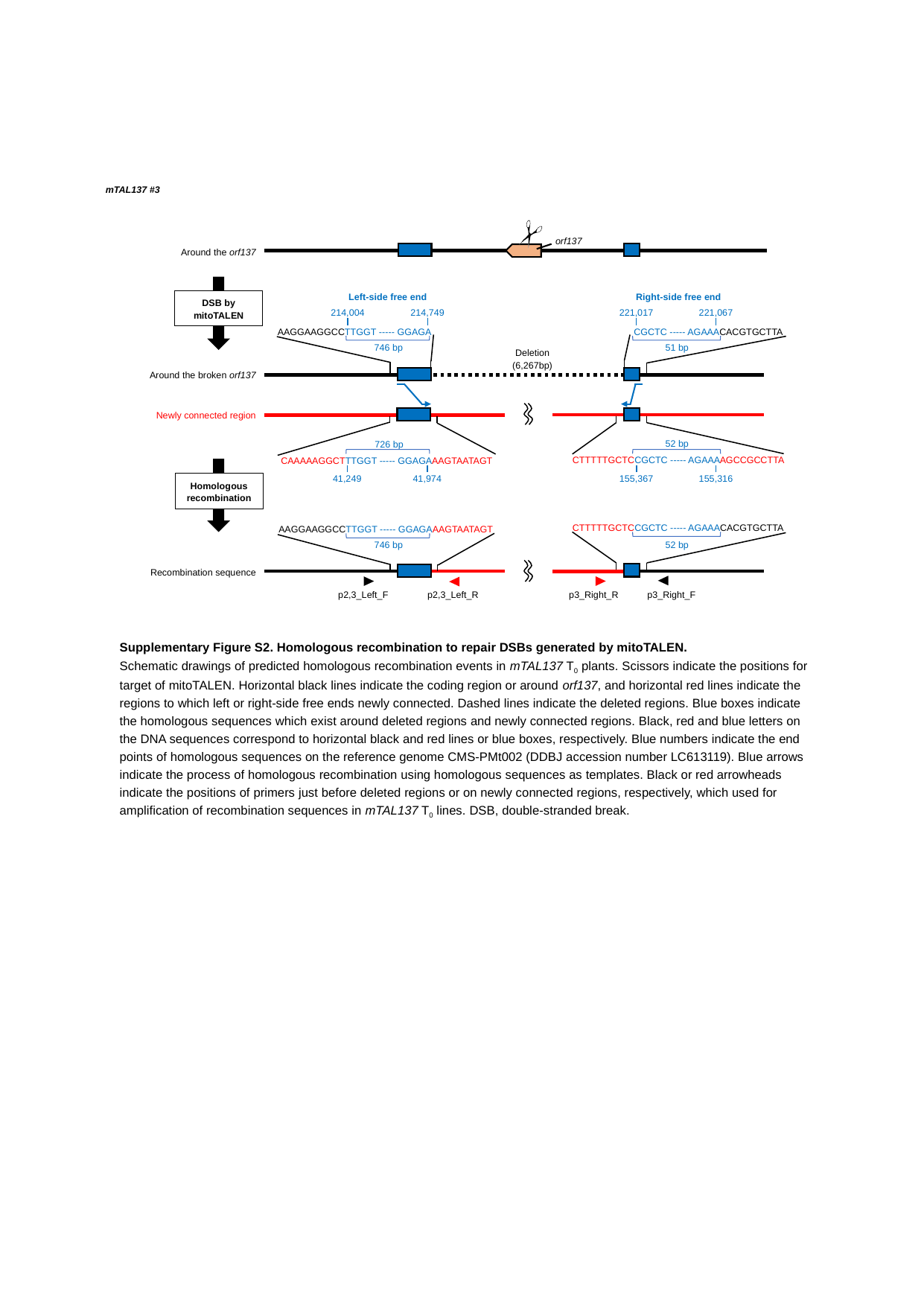

mTAL137 #3
orf137
Around the orf137
Left-side free end
Right-side free end
DSB by
mitoTALEN
214,004
214,749
221,017
221,067
AAGGAAGGCCTTGGT ----- GGAGA
CGCTC ----- AGAAACACGTGCTTA
746 bp
51 bp
Deletion
(6,267bp)
Around the broken orf137
Newly connected region
52 bp
726 bp
CTTTTTGCTCCGCTC ----- AGAAAAGCCGCCTTA
CAAAAAGGCTTTGGT ----- GGAGAAAGTAATAGT
41,249
41,974
155,367
155,316
Homologous
recombination
CTTTTTGCTCCGCTC ----- AGAAACACGTGCTTA
AAGGAAGGCCTTGGT ----- GGAGAAAGTAATAGT
52 bp
746 bp
Recombination sequence
p2,3_Left_F
p2,3_Left_R
p3_Right_R
p3_Right_F
Supplementary Figure S2. Homologous recombination to repair DSBs generated by mitoTALEN.
Schematic drawings of predicted homologous recombination events in mTAL137 T0 plants. Scissors indicate the positions for target of mitoTALEN. Horizontal black lines indicate the coding region or around orf137, and horizontal red lines indicate the regions to which left or right-side free ends newly connected. Dashed lines indicate the deleted regions. Blue boxes indicate the homologous sequences which exist around deleted regions and newly connected regions. Black, red and blue letters on the DNA sequences correspond to horizontal black and red lines or blue boxes, respectively. Blue numbers indicate the end points of homologous sequences on the reference genome CMS-PMt002 (DDBJ accession number LC613119). Blue arrows indicate the process of homologous recombination using homologous sequences as templates. Black or red arrowheads indicate the positions of primers just before deleted regions or on newly connected regions, respectively, which used for amplification of recombination sequences in mTAL137 T0 lines. DSB, double-stranded break.

## Slide 5
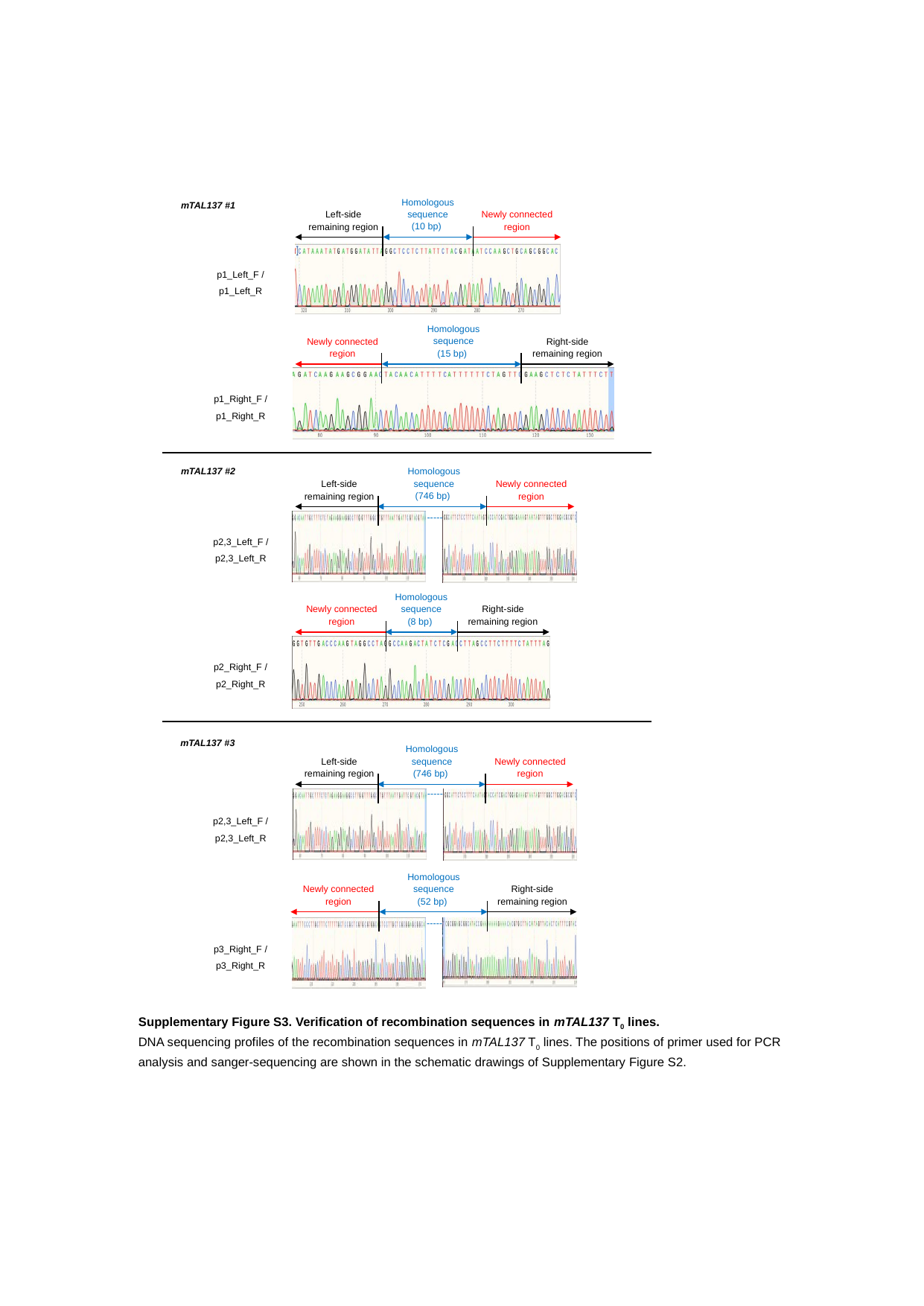

Homologous
sequence
(10 bp)
mTAL137 #1
Left-side remaining region
Newly connected region
p1_Left_F / p1_Left_R
Homologous
sequence
(15 bp)
Newly connected region
Right-side remaining region
p1_Right_F / p1_Right_R
mTAL137 #2
Homologous
sequence
(746 bp)
Left-side remaining region
Newly connected region
-----
p2,3_Left_F / p2,3_Left_R
Homologous
sequence
(8 bp)
Newly connected region
Right-side remaining region
p2_Right_F / p2_Right_R
mTAL137 #3
Homologous
sequence
(746 bp)
Left-side remaining region
Newly connected region
-----
p2,3_Left_F / p2,3_Left_R
Homologous
sequence
(52 bp)
Newly connected region
Right-side remaining region
-----
p3_Right_F / p3_Right_R
Supplementary Figure S3. Verification of recombination sequences in mTAL137 T0 lines.
DNA sequencing profiles of the recombination sequences in mTAL137 T0 lines. The positions of primer used for PCR analysis and sanger-sequencing are shown in the schematic drawings of Supplementary Figure S2.

## Slide 6
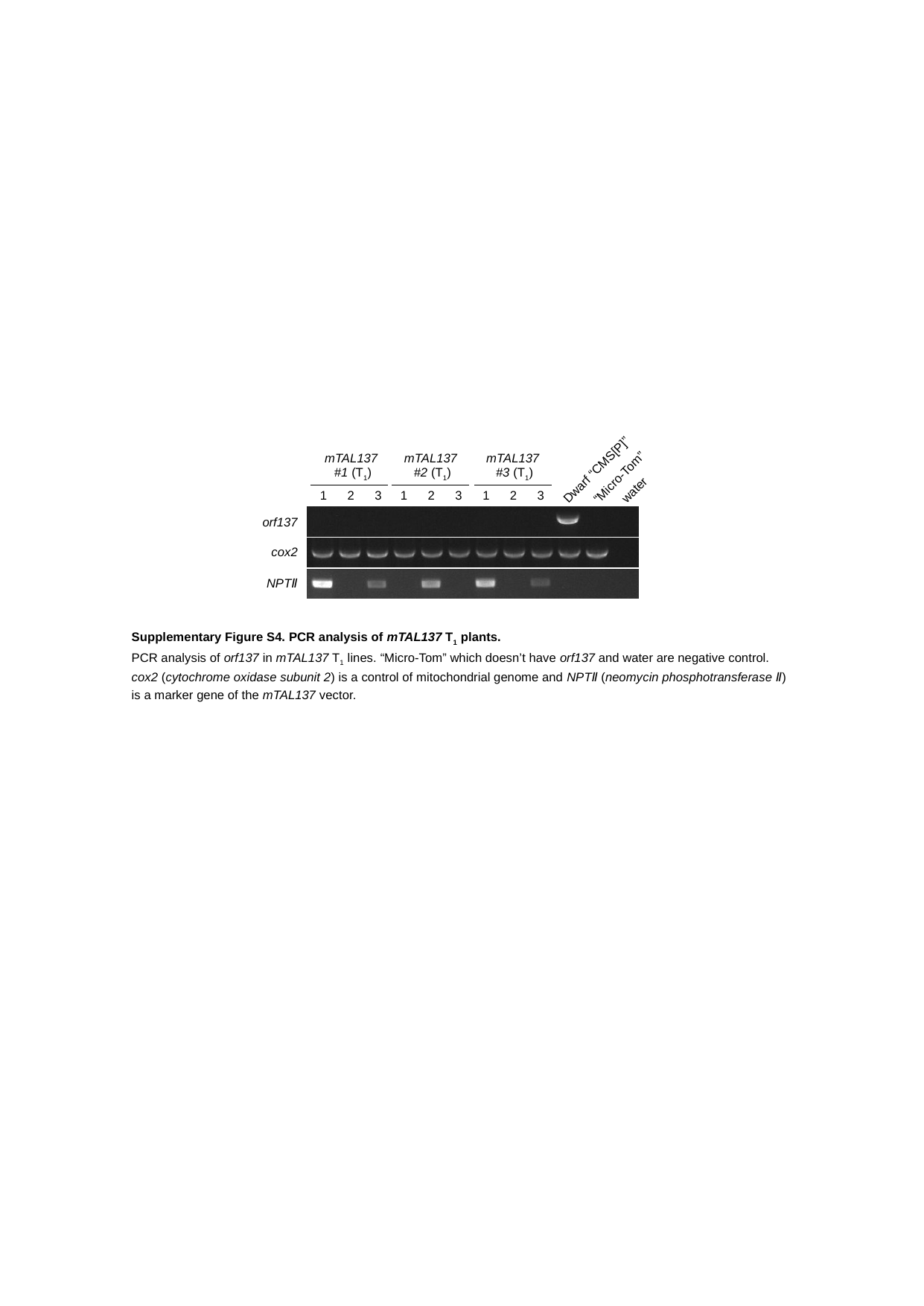

mTAL137
#1 (T1)
mTAL137
#2 (T1)
mTAL137
#3 (T1)
Dwarf “CMS[P]”
“Micro-Tom”
water
3
3
3
2
2
2
1
1
1
orf137
cox2
NPTⅡ
Supplementary Figure S4. PCR analysis of mTAL137 T1 plants.
PCR analysis of orf137 in mTAL137 T1 lines. “Micro-Tom” which doesn’t have orf137 and water are negative control. cox2 (﻿cytochrome oxidase subunit 2) is a control of mitochondrial genome and NPTⅡ (neomycin phosphotransferase Ⅱ) is a marker gene of the mTAL137 vector.

## Slide 7
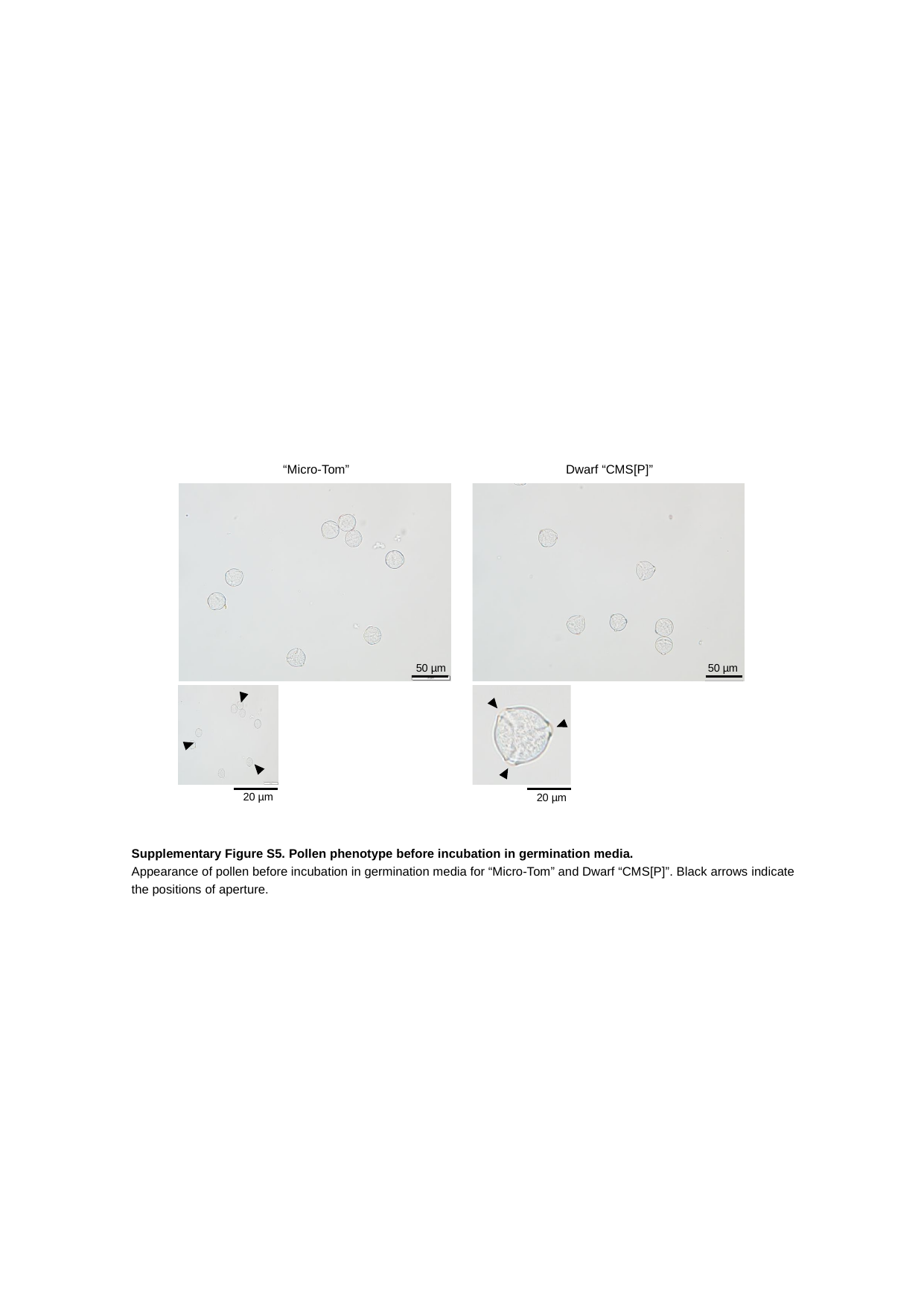

“Micro-Tom”
Dwarf “CMS[P]”
50 µm
50 µm
20 µm
20 µm
Supplementary Figure S5. Pollen phenotype before incubation in germination media.
Appearance of pollen before incubation in germination media for “Micro-Tom” and Dwarf “CMS[P]”. Black arrows indicate the positions of aperture.
