# Supplementary material for: orf137 triggers cytoplasmic male sterility in tomato
Source: Plant Physiol. 2022 Feb 25;189(2):465–8. doi: 10.1093/plphys/kiac082 (PMC9157052; doi:10.1093/plphys/kiac082)
Supplement: kiac082_Supplementary_Data [file kiac082_supplementary_data.zip › 20220303_Kuwabara_Supplementary.docx]

**SUPPLEMENTAL INFORMATION**

***orf137* triggers cytoplasmic male sterility in tomato**

Kosuke Kuwabara^1,2^, Shin-ichi Arimura^3^, Kenta Shirasawa^4^ and Tohru Ariizumi^1,5,*^

^1^ Graduate School of Life and Environmental Sciences, University of Tsukuba, Tsukuba, Ibaraki 305-8577, Japan

^2^ Japan Society for the Promotion of Science (JSPS)

^3^ Graduate School of Agricultural and Life Sciences, University of Tokyo, Bunkyo, Tokyo 113-8654, Japan

^4^ Kazusa DNA Research Institute, Kisarazu, Chiba 292-0818, Japan

^5^ Tsukuba Plant Innovation Research Center, Tsukuba, Ibaraki 305-8577, Japan

*, Corresponding author

**LIST OF SUPPLEMENTAL INFORMATION**

**Methods**

**Data availability statement**

**Supplementary Table S1. Oligonucleotide sequences of PCR primers.**

**Supplementary Figure S1. Mitochondrial gene locations around the region of *orf137*.**

**Supplementary Figure S2. Homologous recombination to repair DSBs generated by mitoTALEN.**

**Supplementary Figure S3. Verification of recombination sequences in *mTAL137* T_0_ lines.**

**Supplementary Figure S4. PCR analysis of *mTAL137* T_1_ plants.**

**Supplementary Figure S5. Pollen phenotype before incubation in germination media.**

**References**

**METHODS**

**Plant materials**

Dwarf “CMS[P]” was developed from “CMS[P]” by backcrossing with tomato dwarf cultivar “Micro-Tom” (TOMJPF0001) (Scott and Harbaugh., 1989) in our previous study (Kuwabara et al., 2021). Dwarf “CMS[P]” was used for *Agrobacterium* transformation. All plants were cultivated at 25 °C under a 16/8 h light/dark cycle.

**Vector construction**

The selection of the target sequence of mitoTALEN and its expression vector construction has been described by Kazama et al. (2019) and Arimura (2022). In brief, target sequences of TALEN in *orf137* were listed using the Old TALEN Targeter (https://tale-nt.cac.cornell.edu/node/add/talen-old). The selected sequences (5′- TTGCAAAGAGCGTTGGA-3′ and 5′- TGTACAGGCAATTGC-3′) were specifically verified to bind any other sites in the mitochondrial genome by BLAST search. The Ti plasmid vector expressing both right and left pair ORFs of TALEN with a mitochondrial targeting signal (Figure 1A) was constructed in two steps. The first step was to make DNA binding domains of the right and left proteins of mitoTALENs specific for the targets by using the Platinum Gate Assembly kit provided by Addgene, according to its protocol (Sakuma et al., 2013); however, the final cloning vectors of the left and right were replaced by pENTR E1 L1-L4 and pENTR E3 L3-L2 of our system ( #158728-#158731 and any of #158721-#158735, Addgene). The resultant left and right ORFs of TALENs were cloned into Ti plasmids by multisite Gateway LR reaction (Thermo Fisher Scientific) with pDESTpK7WG2mito (a Ti plasmid, #158726 in Addgene) and pENTR E2 HSPter-35Sp-mito (#158727 in Addgene, having a terminator of left TALEN ORF, and a two-part promoter and mitochondrial localization signal for right TALEN).

**Tomato transformation**

*The Agrobacterium*-mediated transformation method (Sun et al., 2006) was used for tomato transformation. The cotyledons of Dwarf “CMS[P]” were co-cultivated with *Agrobacterium* strain GV2260 containing mitoTALEN plasmid, and the transformed plants were selected by media containing kanamycin. T_0_ plants were selected via PCR analysis using primers targeting *neomycin phosphotransferase II* (*NPTII*; Table S1).

**DNA extraction, PCR analysis and Sanger sequencing**

Total DNA was extracted from the young leaves using a Maxwell 16 instrument and Maxwell 16 Tissue DNA Purification Kit (Promega). The PCR mixture (10 µL) comprised 0.5 µL total DNA, 0.5 µM primers (Table S1), and 1× GoTaq Green Master Mix (Promega). Amplification was performed under the following thermal cycling conditions: initial denaturation at 95 °C for 2 min; 35 cycles of denaturation at 95 °C for 30 s, annealing at 58 °C for 30 s, and extension at 72 °C for 2 min; and a final extension at 72 °C for 5 min. PCR products were separated via electrophoresis on a 1% agarose gel with TAE buffer. Gels were stained with Midori Green Advance (NIPPON Genetics) to detect the DNA bands under ultraviolet illumination. PCR products were extracted from gels using FastGene Gel/PCR Extraction Kit (NIPPON Genetics) and subjected to Sanger sequencing (Eurofins Genomics). The data were visualized by SnapGene Viewer v6.0 (https://www.snapgene.com).

**Analysis of PacBio long-read sequences**

SMRT sequence libraries, in which each sample was labeled with Barcoded Overhang Adapter Kit (PacBio, Menlo Park, CA, USA), were constructed with an SMRTbell Express Template Prep Kit 2.0 (PacBio) and used for sequencing on a PacBio Sequel IIe system (PacBio) in circular consensus sequencing (CCS) mode. Highly accurate single-molecule consensus (HiFi) reads were obtained from three T_0_ lines of *mTAL137* lines (*#1*, *#2*, and *#3*), and the HiFi reads were mapped on the mitochondrial reference genome of “CMS[P]” (Kuwabara et al., 2021) by minimap2 and mapping status was visualized by IGV. To detect the newly connected regions of transgenic plants, long-read sequences that covered the deleted regions were collected, and the sequences of the connected regions were determined using BLAST (Altschul et al., 1990). PacBio reads from “CMS[P]” were previously obtained (Kuwabara et al., 2021) and were used for mapping and visualization by IGV.

**Pollen germination**

For the *in vivo* pollen germination test on stigma, pistils were fixed in ethanol: acetic acid (3: 1, v/v) solution 24 h after self-pollination. Fixed pistils were soaked in 5 M NaOH for 24 h. After washing thrice, the pistils were stained with 0.001 w/v% aniline blue in 0.1 M K_2_HPO_4_ buffer (pH 10) for 24 h in the dark. Pollen tubes were observed using a BX53 microscope (Olympus), and digital images were captured using a DP72 camera (Olympus).

The pollen germination test using liquid germination medium was performed according to a previously described protocol with few modifications (Steven and Wouter, 1993). Briefly, freshly opened flowers (3 flowers per 1.5 mL tube) were soaked in 1 mL of germination media containing 15.1 w/v% polyethylene glycol (average molecular weight: 6000), 10 w/v% sucrose, 1.63 mM H_3_BO_3_, 1.27 mM Ca(NO_3_)_2_, 1 mM MgSO_4_, 1 mM KNO_3_, and 0.1 mM K_2_HPO_4_; the mixture was strongly vortexed to release the pollen from anthers. After removal of flower residues, the pollen suspension was incubated in the germination medium and agitated using a rotator at 25 °C for 4 h. Thereafter, the pollen tubes were observed under a BX53 microscope (Olympus). The ratios of the germinated or abnormal pollen were calculated using three biological replicates of the independent experiments with more than 300 pollen grains observed for each experiment.

**DATA AVAILABILITY STATEMENT**

The data that support the findings of this study and Materials used in this study are available from the corresponding author upon reasonable request.

**REFERENCES**

**Altschul, S. F., Gish, W., Miller, W., Myers, E. W., and Lipman, D. J.** (1990). Basic local alignment search tool. *J. Mol. Biol.* **215**:403–410.

**Arimura, S.** (2022). MitoTALENs: A method for targeted gene disruption in plant mitochondrial genomes. *Methods Mol. Biol.* **2363**: 335-340.

**Kazama, T., Okuno, M., Watari, Y., Yanase, S., Koizuka, C., Tsuruta, Y., Sugaya, H., Toyoda, A., Itoh, T., Tsutsumi, N., et al.** (2019). Curing cytoplasmic male sterility via TALEN-mediated mitochondrial genome editing. *Nat. Plants* **5**:722–730.

**Kuwabara, K., Harada, I., Matsuzawa, Y., and Ariizumi, T.** (2021). Organelle genome assembly uncovers the dynamic genome reorganization and cytoplasmic male sterility associated genes in tomato. *Hortic. Res.* **8**:1–11.

**Sakuma, T., Ochiai, H., Kaneko, T., Mashimo, T., Tokumasu, D., Sakane, Y., Suzuki, K. I., Miyamoto, T., Sakamoto, N., Matsuura, S., et al.** (2013). Repeating pattern of non-RVD variations in DNA-binding modules enhances TALEN activity. *Sci. Rep.* **3**:3379.

**Steven, P. C. G., and Wouter, de R.** (1993). Stimulation of tomato pollen germination by the flavonoid quercetin. *Rep. Tomato Genet. Coop.* **43**:19–20.

**Sun, H. J., Uchii, S., Watanabe, S., and Ezura, H.** (2006). A highly efficient transformation protocol for Micro-Tom, a model cultivar for tomato functional genomics. *Plant Cell Physiol.* **47**:426–431.
